# Systemic Cardiac Complications of Topical Timolol: A Case of Advanced Heart Block Requiring Pacemaker Implantation

**DOI:** 10.51894/001c.144467

**Published:** 2025-09-30

**Authors:** Sai Tharun R. Gopannagari

## 84

### INTRODUCTION

Timolol, a non-selective beta-blocker used in open-angle glaucoma management acts by lowering IOP. Topical Timolol is generally considered safe, however, due to its systemic absorption, it can lead to significant cardiovascular complications such as Bradycardia, Heart blocks, Syncope, and Congestive Heart failure. We present a case of Timolol-triggered second-degree AV block type 1 and third-degree AV block leading to presyncope requiring permanent pacemaker placement in a patient who has been using topical Timolol, demonstrating the necessity for awareness of systemic effects even with topical therapy.

### CASE PRESENTATION

A 90-year-old male with a past medical history of Diabetes Mellitus, Hypertension, CKD, and Glaucoma was admitted for the evaluation of Fall. He mentioned that he felt dizzy when he got up from the chair and fell back but did not lose consciousness. His home medications include Amlodipine, Brimonidine-Timolol 0.2-0.5%, Dorzolamide-Timolol 2-0.5% ophthalmic solution, and Latanoprost. He has been taking Timolol eye drops since 2020. Initial EKG showed LBBB, Occasional PVCs, 1st-degree heart block, and a Heart rate of 59. Overnight telemetry was reviewed, showing second-degree AV block type 1 and third-degree AV block. His Timolol eye drops were withheld. A DDD/ CLS permanent pacemaker with HR ranging between 60-120 bpm was implanted. Repeat EKG showed pacemaker spikes, LBBB, and HR of 67. Hospital course post-pacemaker implantation was uneventful with HR ranging between 60-70s.

### DISCUSSION

Since introducing beta-blockers in treating Glaucoma, they have become the most often prescribed drugs. Topical Timolol has systemic absorption up to 80% and can produce side effects such as bradycardia, arrhythmia, congestive heart failure, syncope, and heart block. Our case shows that in a 90-year-old patient, who might have a degenerative component of AV Block, Timolol acting as an additional inciting factor led to second-degree AV block type 1 and third-degree AV block consequently leading to Presyncope requiring a permanent pacemaker. We recommend that elderly patients who are taking Timolol eye drops should be closely monitored for cardiovascular side effects such as heart blocks and symptomatic bradycardia, and consider other alternative options for Glaucoma treatment.

